# Dual-scale single marker calibration for digital templating of total hip arthroplasty in standing radiographs: a prospective clinical study

**DOI:** 10.1007/s00402-022-04355-y

**Published:** 2022-01-31

**Authors:** Christian Ries, Christoph Tobias Baltin, Stefan Haneder, Peer Eysel, Martin Hellmich, Christoph Kolja Boese

**Affiliations:** 1 Department of Orthopaedic and Trauma Surgery, University Hospital of Cologne, Joseph-Stelzmann-Str. 24, 50931 Cologne, Germany; 2 Division of Orthopaedics, Department of Trauma and Orthopaedic Surgery, University Medical Center Hamburg-Eppendorf, Martinistr. 52, 20246 Hamburg, Germany; 3 Department of Diagnostic and Interventional Radiology, University Hospital Cologne, Kerpener Str. 62, 50931 Cologne, Germany; 4 IMSB, University of Cologne, Kerpener Str. 62, 50937 Cologne, Germany

**Keywords:** Hip arthroplasty, Digital radiography, Digital templating, Hip replacement

## Abstract

**Purpose:**

Accuracy of calibration of radiographs significantly influences the quality of digital templating for total hip arthroplasty (THA). The standard of care is calibration with external calibration markers (ECM). This method is associated with significant errors. Dual-scale single marker (DSSM) calibration methods may improve accuracy. The present prospective observational study is the first to analyze the application of a DSSM method in standing pelvis radiographs.

**Methods:**

100 patients with unilateral THA underwent antero-posterior pelvis radiographs with ECM and DSSM. The hip components were used as reference calibration factor (internal calibration factor; ICM). Absolute differences of calibration factors for ECM and DSSM from ICM were calculated. Absolute relative deviations (ARD) were calculated. Subgroup analysis for sex and WHO BMI category was performed. Furthermore, patients reported subjective comfort for each marker using a 10-point scale and choosing the preferred marker.

**Results:**

Maximum magnification factor differences from the ICM were 23.3% and 9.5% and mean absolute differences were 12.5% and 2.1% for the ECM and DSSM, respectively. ARD from ICM was significantly lower for DSSM compared to ECM (*p* < 0.001). Absolute differences increased with BMI category using ECM; calibration by DSSM was consistent in all subgroups. Patients preferred DSSM over ECM (*n* = 53) or were indifferent (*n* = 20). Comfort was rated significantly higher for DSSM versus ECM (*p* < 0.001).

**Conclusion:**

DSSM method showed superior results in comparison to the ECM method for calibration of digital radiographs. DSSM could be used to improve digital templating in standing radiographs.

## Background

In total joint replacement, digital templating is performed as standard of care [1, 2]. Here, precise calibration of the hip plane is required to correctly select implant sizes [1]. Spherical radio-opaque external calibration markers (ECM) are the established standard. However, they have been shown to result in significant calibration errors [1, 3–6]. This might subsequently lead to erroneous templating [6]. Alternatively, using fixed calibration factors (FCF) was discussed [1, 7, 8]. Notably, these methods do not account for patient positioning relative to the X-ray detector or individual anatomy. The application of dual-scale calibration markers was introduced by King et al. to identify the patient’s sagittal diameter and subsequently calculate the hip center position [9]. The assumed hip plane is calculated using computed tomography-based reference values. While calibration of the hip in standing antero-posterior radiographs of the pelvis is the standard for digital templating, the method of King et al. has only been applied to supine radiographs [10]. Recently, a dual-scale single marker (DSSM) method was published for supine radiographs [11]. Here, the sagittal diameter of the patient can be deducted with a single marker in front of the pelvis and the given setup of the X-ray construction.

This study aimed to compare a DSSM method to a reference marker in standing antero-posterior radiographs of the pelvis. The reference marker was an internal calibration marker (i.e., unilateral THA component; ICM). Additionally, a standard single external calibration marker (ECM) was compared to the DSSM method.

The DSSM method to calculate the hip plane and corresponding calibration factor is based on a sample of 400 pelvic computed tomographies. It has been shown to be superior to mono-marker methods and fixed calibration factors in a simulation as well as in supine radiographs [7, 11].

## Material and methods

A prospective clinical study of 100 patients was performed. All patients who received standing antero-posterior radiographs of the pelvis for any reason were screened for eligibility at a single arthroplasty center. No radiographs were taken without clinical indication. Inclusion criteria were as follows: unilateral THA, known implant sizes, informed consent, “Low” standing pelvic radiograph, and documentation of height and weight.

**Fig. 1:**
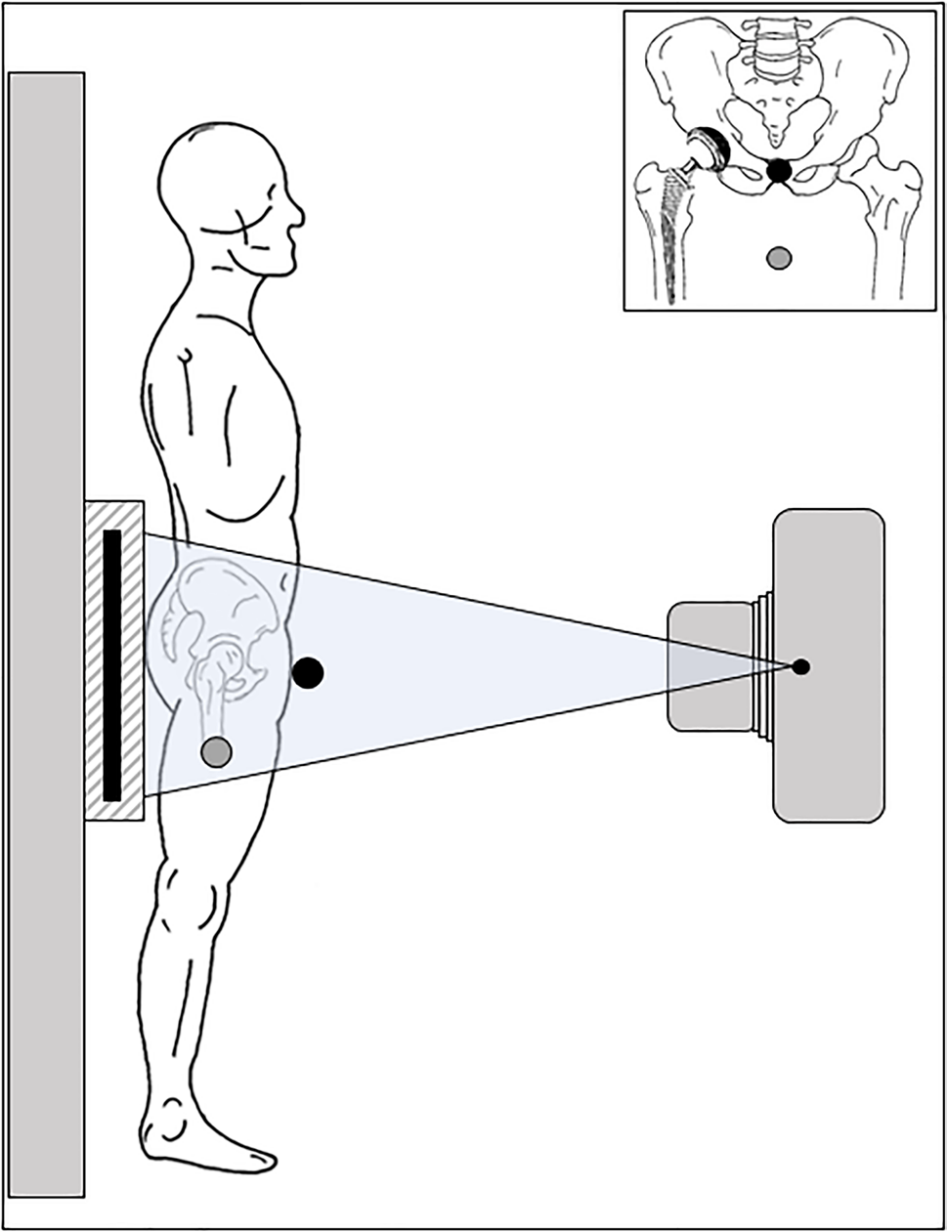
Placement of anterior DSSM marker ball at belt-buckle position (black circle) and standard marker ball between the legs (gray circle) in standing radiograph setup. A corresponding radiograph sketch is demonstrated in the top right corner

Marker placement was performed following the instructions for use. Two types of external calibration markers were attached: (1) DSSM at “belt-buckle” position in front of the pubic symphysis; (2) standard external marker between the patients’ legs (Fig. 1).

Exclusion criteria were as follows: refusal of consent, incomplete information on THA component size, technical incorrect radiograph (following standard rules), external radiographs (imported radiographs from other sources), missing external markers, obviously mal-positioned external marker (e.g., in front of the thigh, lateral of the thigh), incomplete documentation, anatomical deformities of the pelvis (e.g., bone tumors, anatomical variations, post-operative changes (except for THA), fractures of the pelvis (new or old), foreign material in the pelvis (except for THA/markers), markers not completely visible, and radiographs taken under restrictions impeding correct radiographs to be taken.

The age minimum was 18 years with no maximum age.

This study was registered in the German Clinical Trials Register (DRKS00012844). This study was approved by the local ethics committee (16-275).

All data were pseudonymized. Sex, height in cm, weight in kg, and waist circumference at the height of the anterior marker in cm were documented. BMI was calculated and stratified according to the WHO classification. Patients were asked to complete a questionnaire of three questions regarding the comfort of DSSM and ECM independently and in comparison. The subjective comfort scale asked the patient to score one to ten points on a visual numeric scale per marker. A value of “one” represented significant discomfort by marker/placement and “ten” no discomfort at all. Question one asked about the ECM, question two the DSSM. The third question asked which marker the patient preferred (ECM, DSSM, or indifferent).

All radiographs were stored in a picture archiving and communication system (PACS). Measurements were performed using a proprietary DICOM viewer (IMPAXX EE). The outer circumference of all markers was identified by three points. The diameter was measured in mm with one decimal. Repeated measurements of all radiographs were performed by one experienced observer. If two results differed, a third measurement was performed. The mean of the two best fits was used. The following markers were measured in the same order in all radiographs: (1) DSSM, (2) ICM (femoral head component and acetabular component; the best visible marker was marked and used for the analysis), and (3) the ECM.

Calculation of calibration factors (CF) in percent was performed for each marker separately. The CF of the ICM and ECM was calculated using the standard method following formula:

1 $${\text{CF}} = {{{\text{Measured}}\,{\text{diameter}}} \mathord{\left/ {\vphantom {{{\text{Measured}}\,{\text{diameter}}} {{\text{True}}\,{\text{diameter}}}}} \right. \kern-\nulldelimiterspace} {{\text{True}}\,{\text{diameter}}}}$$

The calibration factor is without unit. By multiplication with 100, the magnification factor of a radiograph can be calculated (reported as percentage magnification).

For the DSSM method, the patient’s sagittal diameter was calculated by the calculated position of the anterior DSSM over the detector plane and the surface plane of the wall bucky stand. The latter being considered to be the posterior limit of the patient. The height of the DSSM above the detector in mm was calculated by the formula presented by Boese et al. [7]. The distance of the standing wall bucky surface to the detector plane was measured with a flat marker placed directly on the surface of the wall bucky in 100 standing radiographs. The distance from the detector plane was calculated using the intercept theorem. The median distance of the marker to the detector plane was 68 mm. Subtracting the marker height of 5 mm, the surface detector distance was defined as 63 mm.

The difference of the DSSM height above the detector minus 63 mm was identified as the patient’s sagittal diameter. The supposed height of the hip center above the detector was calculated following the sex-specific linear regression model by Boese et al. [7]. Here, the linear model predicts an empirical hip plane position above the detector based on the patient’s sagittal diameter and sex.

Repeated measurements of the markers were performed by two independent observers blinded to the previous results in a subset of 10 radiographs. The first 10 radiographs were chosen to limit potential selection bias.

### Sample size calculation

On the basis of the X-rays of 100 patients, the following characteristics were determined retrospectively for the absolute relative deviation (ARD) distribution to compare ICM and ECM: mean value 0.046; standard deviation 0.043; minimum 0.001; 25th percentile 0.015; median 0.031; 75th percentile 0.063; and maximum 0.214. With the cautious assumption of a standard deviation of 0.05 and a correlation of 0.5 between the paired measurement series for ECM and DSSM, 90 patients/X-rays are required to detect a position difference of 0.015 with power 80% using the paired *t*-test (two-sided level of significance 5%; Stata/SE 13.1, StataCorp, College Station, TX, USA, power paired means). In order to compensate for the possibly lower power of the Wilcoxon signed rank test, a total of 100 patients should be prospectively examined.

### Statistical analysis

Quantitative variables were summarized as mean ± standard deviation, median, and range, qualitative variables by absolute and relative (%) frequencies. Frequency distributions are visualized by box-and-whiskers plots and histograms. Pearson's correlation coefficient [with two-sided *p*-value for the test against (0)] was calculated to describe the strength of linear relationship of any two variables of interest. The primary comparison of the absolute relative deviations (ARD) of methods ECM and DSSM

- (i) ARD-DSSM = absolute (CF-DSSM − CF-ICM)/CF-ICM,
- (ii) ARD-ECM = absolute (CF-ECM − CF-ICM)/CF-ICM,

was evaluated by the (paired) Wilcoxon signed rank test at two-sided significance level 5%. Box-plots were generated. Calculations were done with SPSS Statistics (IBM Corp., Armonk, NY, USA).

Intra-class correlation coefficients with 95% confidence intervals were calculated. A mixed model with absolute agreement was chosen.

### Primary outcome parameter

H_0_: No difference in the distribution of ARD of the ECM and the DSSM.

## Results

### Demographic baseline characteristics

Per protocol, 100 subjects were enrolled and qualified for inclusion. 52 were female. In 55 cases, the acetabular component was used as ICM, in 45 cases the femoral head component.

Patient mean height and weight were 171 cm (SD 11; range 145–196) and 83.2 kg (SD 16.0; range 42–133), respectively. The mean BMI was 28.3 kg/m^2^ (SD  4.6; range 17.3–41.0). Frequencies of WHO BMI categories were as follows: 1 underweight, 26 normal weight, 37 pre-obesity, 28 obesity class I, 7 obesity class II, and 1 obesity class III. The mean waist circumference at the height of the anterior marker was 106 cm (SD 10.6; range 78–135).

### Patient reported preferred marker

The DSSM and the ECM received an average rating of 9.2 (SD 1.3; range 5–10) and 8.7 (SD 1.8; range 4–10) on the subjective comfort scale, respectively. The difference was significant (*p* < 0.001). Twenty-seven patients preferred the ECM, 53 preferred the DSSM, and 20 were indifferent. For those preferring the ECM, many reported extensive palpation of the pubic symphysis when positioning the anterior DSSM by the radiology team members contrary to instructions for use.

### Calibration factors

**Fig. 2:**
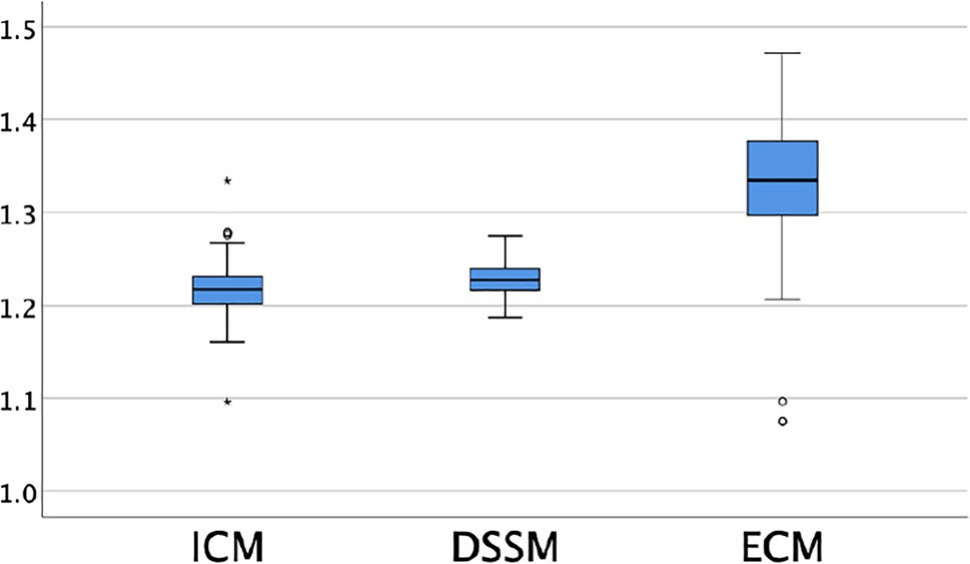
Box-plots of calibration factors for ICM, ECM, and DSSM. Circles represent outliers, asterisks extreme outliers. Calibration factors have no unit

The mean reference internal calibration factor (ICM) was 1.217 (SD 0.029; range 1.096–1.334). The mean ECM calibration factor was 1.334 (SD 0.075; range 1.075–1.471), the mean DSSM calibration factor was 1.229 (SD 0.017; range 1.187–1.275). Figure 2 shows the distributions of the calibration factors.

The absolute differences for two methods and the reference value, ICM, are shown in Table 1. The median absolute difference of the magnification factor (100 times the calibration factor as percent) from the reference ICM was 12.9% for the ECM and 1.7% for the DSSM. The absolute relative deviations are presented in Table 1. The difference between ECM and DSSM was significant (*p* < 0.001).

**Table 1:** Absolute differences for the two methods and the reference value, ICM, and the absolute relative deviation.

Marker	Absolute differences from ICM					Absolute relative deviation (ARD)				
	Median	Mean	SD	Min	Max	Median	Mean	SD	Min	Max
DSSM	0.017	0.021	0.020	0.000	0.095	0.013	0.018	0.016	0.000	0.087
ECM	0.129	0.125	0.055	0.002	0.233	0.105	0.103	0.045	0.002	0.189

Values are given as calibration factor without unit

### Distribution and frequencies of error

**Fig. 3:**
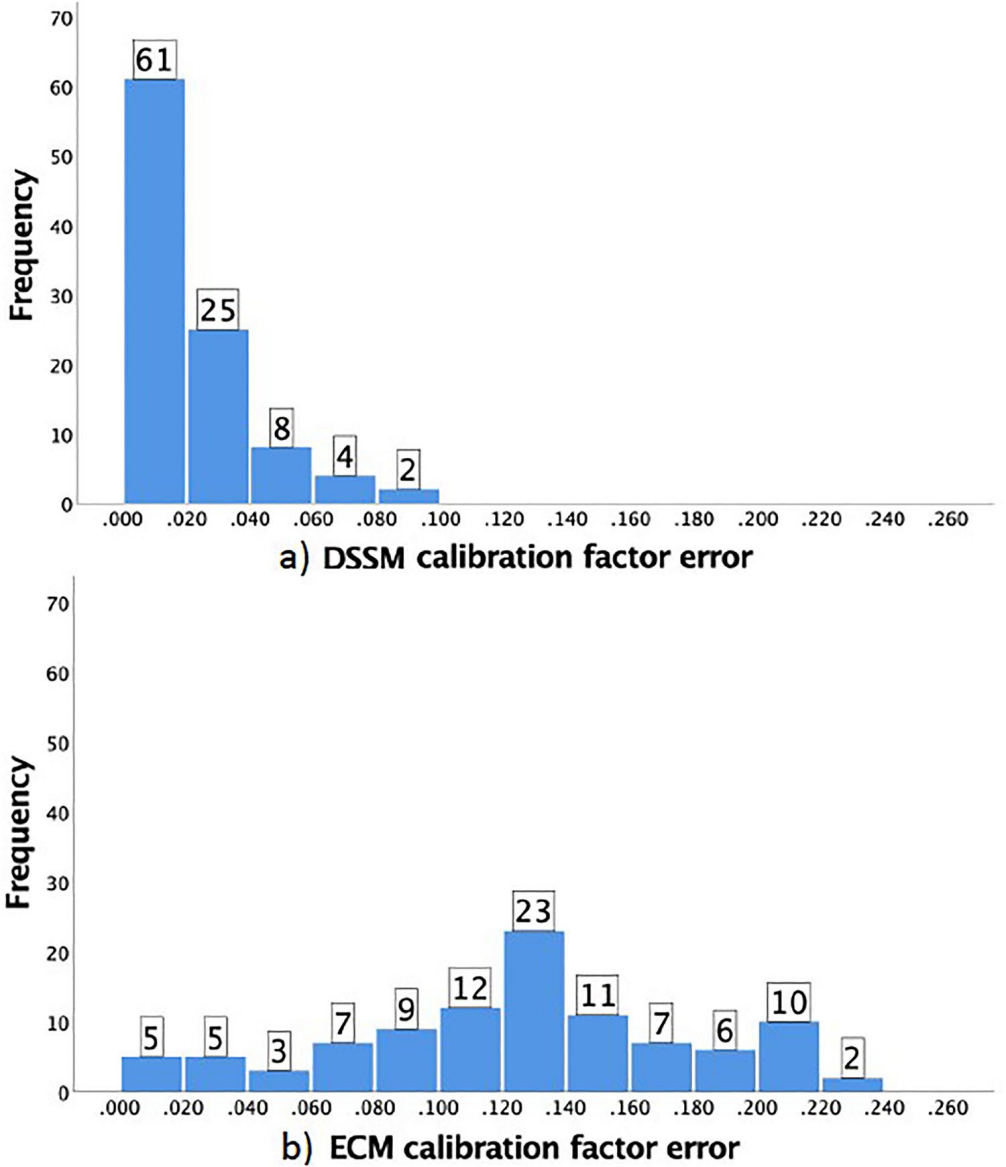
Absolute difference from ICM frequencies of **a** DSSM and **b** ECM in 0.02 calibration factor increments (e.g., 0–1.999, 2.000–3.999, etc.)

The frequency of calibration factor errors above 10% from the ICM was reduced from 71 to 0 by the DSSM method. For the DSSM, 61% of the differences were equal to or below 2% error and 76% were equal to or below 4% error (Fig. 3).

### Subgroup analysis by sex and BMI

**Fig. 4:**
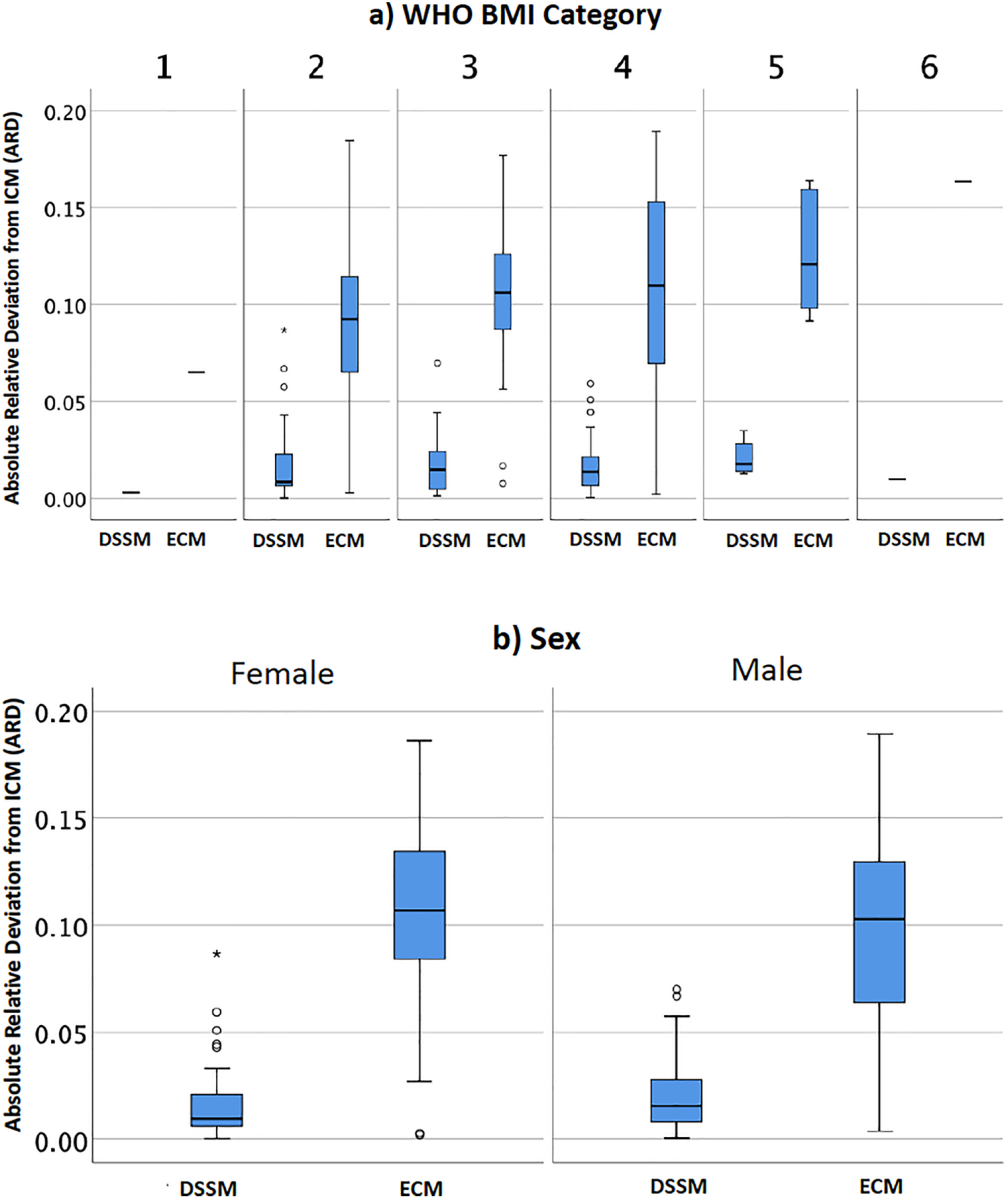
Absolute relative deviation (ARD) of ECM and DSSM method from ICM reference. **a** Stratification by BMI category. Underweight and obesity class III included only one case. **b** Stratification by sex

ARD of DSSM showed no correlation with BMI (*r* = 0.020, *p* = 0.847); ECM showed a weak positive correlation (*r* = 0.266, *p* = 0.007). Fig. 4a, b depicts the ARD for DSSM and ECM by BMI category and sex.

The BMI effect on the calibration methods is shown in Table 2 for absolute differences of the calibration factors.

**Table 2:** Absolute differences of ECM and DSSM method from ICM reference

BMI category	*N*	DSSM					ECM				
		Mean	Median	SD	Min	Max	Mean	Median	SD	Min	Max
Underweight	1	0.003	0.003	–	0.003	0.003	0.077	0.077	–	0.077	0.077
Normal weight	26	0.022	0.010	0.025	0.000	0.095	0.106	0.112	0.052	0.004	0.214
Pre-obesity	37	0.021	0.018	0.018	0.001	0.093	0.130	0.129	0.047	0.009	0.215
Obesity class I	28	0.021	0.017	0.019	0.000	0.071	0.129	0.135	0.066	0.002	0.233
Obesity class II	7	0.026	0.022	0.011	0.015	0.042	0.157	0.147	0.042	0.112	0.205
Obesity class III	1	0.012	0.012	–	0.012	0.012	0.198	0.198	–	0.198	0.198

Stratification by BMI category. Underweight and obesity class III included only one case

### Inter- and intra-rater agreement

The intra- and inter-rater correlation for measurement of markers were 0.999 (CI 0.995–1.000) and 0.975 (CI 0.906–0.994) for the DSSM, 0.999 (CI 0.995–1.000) and 0.998 (CI 0.989–1.000) for the ICM (Acetabular component), 1.000 (CI 1.000–1.000) and 1.000 (CI 1.000–1.000) for the ICM (Femoral ball head), and 0.999 (CI 0.996–1.000) and 0.998 (CI 0.992–0.999) for the ECM, respectively.

## Discussion

The role of pre-operative templating in total joint replacement is generally accepted [2, 4]. Additionally, for medico-legal reasons and quality management it is often required [2]. However, the accuracy of calibration with external markers has been questioned in recent publications [1, 3–5, 7, 11–13]. Correct calibration of radiographs is a key requirement for high quality digital templating [4, 8]. Therefore, optimization of radiograph calibration is essential for templating. While positioning of external calibration markers can be optimized to a certain degree, the method relies on manual placement [4, 5]. Alternatively, a fixed calibration factor can be used [14]. However, this will automatically result in errors for all patients whose hips are not in the exact plane of the assumed factor. The dual-scale calibration marker method by King et al. provides an empirical data-based approach less sensitive to manual placement of markers for the calculation of calibration factors [9, 15].

The presented DSSM method has been introduced in supine radiographs and is an advanced version of the King method. Here, the underlying CT-based data for calculation of hip planes is more sophisticated [7]. Secondly, the technical solution can be used in supine as well as in standing pelvis radiographs. This is of particular interest when application of weight bearing radiographs are sought. Additionally, the DSSM method only requires a standard spherical marker without need for a bulky and more complex device as is required for the method by King.

In the present study, post-operative radiographs of patients with unilateral THA were performed with two types of external calibration markers: (1) anterior marker at “belt-buckle” position in front of the pubic symphysis, and a (2) standard external marker between the patients’ legs. The internal hip components were used as individual reference.

Overall, the DSSM method was superior to the ECM method. The mean error of the ECM method as well as the number of outliers could be reduced significantly. Additionally, the DSSM method showed consistent results in all WHO BMI categories and proved to be applicable in non-obese as well as obese patients. While it was not part of the study protocol, it is anticipated that the novel DSSM method is less time consuming for the healthcare professional in comparison to conventional methods or the King method. For the DSSM method, the marker placement is less complex and does not require palpation of anatomical landmarks.

Acceptance of the novel method was high. Overall, patients preferred the DSSM over the ECM method. However, repeated palpation of the pubic symphysis resulted in preference of ECM over DSSM in some cases. Notably, the instructions for the radiology team members and the training do not require palpation of the symphysis. Therefore, it may be assumed that the acceptance for the DSSM can be further improved. Additionally, preferences and ease of use by the application health care professional may be surveyed in future studies.

In a recent study using the DSSM method in supine radiographs, the mean (range) absolute differences of DSSM and ECM were 0.011 (0.056–0.009) and 0.105 (0.002–0.182), each [11]. The present results of 0.021 (0.000–0.095) and 0.125 (0.002–0.233) for DSSM and ECM repeatedly showed the superiority of the DSSM method. However, the deviation of the DSSM from the reference marker was somewhat higher in the present study of standing antero-posterior radiographs. This may be related to the fact that the CT-based reference values for the DSSM method were derived from supine positioned patients and therefore are less reliable in standing radiographs. In supine position, the patient will be as close as possible to the detector and not move significantly during radiography. In standing radiographs, it was not possible to control whether standing patients deviated from the position close to the standing wall bucky and thus may have increased the calculated sagittal patient diameter unintentionally. Still, the results show a very low mean magnification error of 2.1% with the DSSM method.

Based on these results, the supine method may be superior to the standing DSSM method. However, if standing radiographs are required, the new method has shown to provide very good results in comparison to the ECM method and previous reports on alternative methods.

There were limitations to this study. First, the most important limitation may be patient positioning in standing antero-posterior radiographs. The posterior reference was fixed to the table while the patients position relative to the table was not. Therefore, the patient could potentially move away from the table and thus influence the measured sagittal diameter. Consequently, patients were asked to slightly lean against the table to reduce this source of error. Potentially, adaptions to the calculation method are required to address systematical errors. Secondly, better results could be achieved with the ECM method. In this setting, the radiological assistants were trained with all markers simultaneously and repeatedly to achieve comparable quality. Finally, the underlying measurements and calculations are not automated. Future implementation of such a method into common templating software may help improve calibration and templating.

In conclusion, the sex-specific DSSM method showed superior results in comparison to the ECM method. DSSM could be used to improve digital templating in standing radiographs. Additionally, it was consistent in all BMI categories and both sexes.

## Data Availability

Data are stored on file at the University Hospital Cologne.

## Declarations

### Study registration

This study is registered as DRKS00012844 in the German Clinical Trials Registry.
